# Increased anxiety from fear of Omicron in China as compared to North America and Western Europe: A cross-sectional Kendall’s tau-b analysis using the generalized anxiety disorder 7-item questionnaire

**DOI:** 10.3389/fpsyt.2022.977361

**Published:** 2022-08-30

**Authors:** Dan Shan, Chang Liu, Shaoyang Li, Yuandian Zheng

**Affiliations:** ^1^COVID-19 Response Social Organization Collaboration Network, Qujing, China; ^2^School of Clinical Medicine, University of Cambridge, Cambridge, United Kingdom; ^3^Faculty of Science, The Hong Kong Polytechnic University, Hong Kong, Hong Kong SAR, China; ^4^College of Osteopathic Medicine, Kansas City University, Kansas City, MO, United States

**Keywords:** anxiety, COVID-19, Omicron, pandemic, sequelae

## Abstract

**Background:**

Policies dealing with the Coronavirus Disease 2019 (COVID-19) pandemic vary across the globe, the different governmental responses then affect the public perception of COVID-19. Many unofficial Chinese media outlets frequently spread misinformation about COVID-19 and exaggerated reports of rare sequelae of Omicron for monetization and attention seeking, leading to panics in the Chinese public. In comparison the attitudes toward Omicron in other countries around the world, especially in North America and Western Europe have shifted to a more relaxed stance.

**Objective:**

This article primarily aims to investigate the association between Chinese people’s attitudes toward the potential after-effects of Omicron and their anxiety status, as compared to these of people living in North America or Western Europe.

**Methods:**

We conducted a questionnaire survey *via* the Credamo and collected valid data from 500 Chinese (not living in Shanghai), another 500 Chinese (living in Shanghai) and 500 people living in North America or Western Europe in June 2022. Kendall’s coefficient of rank correlation tau-sub-b was used to examine this association.

**Results:**

The results suggested that subjective attitudes of Chinese participants toward the sequelae of Omicron were positively and significantly associated with their anxiety status [i.e., the Generalized Anxiety Disorder 7-item (GAD-7) scores] in Shanghai (China) (*Tb* = 0.44, *p* < 0.01) and other parts of China outside Shanghai (*Tb* = 0.37, *p* < 0.01). However, no such significant correlation was found in North America & Western Europe (*Tb* = -0.01, *p* > 0.05).

**Conclusion:**

Our findings showed that Chinese participants who were more worried about the after-effects of Omicron had higher levels of anxiety. Although it is true that Long COVID-19 should be a concern, exaggerated media reporting can impact negatively on an individual’s mental wellbeing. Only through the dissemination of robust scientific studies, the misinformation and the fears that follow it can be put to rest.

## Introduction

### Background

Coronavirus Disease 2019 (COVID-19) outbreak elicited by severe acute respiratory syndrome coronavirus 2 (SARS-CoV-2) began in December 2019 (1). As of June 7, 2022, the COVID-19 has led to more than 536 million confirmed cases and 6.32 million deaths globally (2). It has been considered a serious event, impacting significantly on the mental health of the global population (3).

### Coronavirus disease 2019 and anxiety

In order to reduce the risk of coronavirus exposure in public, staying-at-home campaign was highly recommended or even mandated (4). As a result, the fear of contracting the virus, high unemployment due to economic loss, interrupted daily routine during recurrent periods of lockdown, the inability of engaging in most canceled outdoor events and other factors induced by COVID-19 severely impacted public mental health (5). In the general Chinese population, varying degrees of anxiety resulted from many factors, such as overestimating threat and intolerance of uncertainty to COVID-19, ranging from 2 to 37%, yielded a pooled prevalence of 19.1% (6–14). In North America and Western Europe, a pooled prevalence of anxiety was slightly lower than 15% (15).

### Omicron and after-effects

Omicron, a newest and most popular variant of the Coronavirus, firstly discovered on November 24, 2021, has clinical characteristics mainly consisting of mild symptoms but extremely high communicable capacity (16). With reference to the after-effects of COVID-19, previous studies showed that delta variant or other preceding variants could possibly cause patients many impacts such as hair loss, altered sense of smell and taste (17–19), while the consequence of Omicron is unclear and still under evaluation (20).

### Omicron in China

The COVID-19 pandemic was well controlled in China owing to its zero-tolerance approach to coronavirus applied in the past 2 years (21–24), but for this reason, no herd immunity barrier has been established (25); meanwhile, other countries (especially Western countries) attempted coexistence with COVID-19 in order to return things that were severely impacted such as economy by pandemic to normal (26). Hence, once the pandemic spread internationally, the potential risk caused by highly contagious Omicron to the whole country (i.e., China) could be very high (27). Unfortunately, Omicron suddenly broke out in Shanghai, China starting in March and continued to grow at a rate of about 10,000 confirmed patients per day until May (28, 29). The dire situation was not brought under control until early June (30, 31).

### People’s perception of coronavirus disease 2019 *via* media outlets in China and the West

It is important to note that all traditional news media outlets in China are controlled by Central Publicity Department (CPD) the information published come under more censorship than their western counterparts (32). As is often the case, alternative forms of traditional media flourished instead under the radar of the government control regime, citizen journalism as it is coined became the new way many people obtain news (33, 34). From this understanding it is evident that the flourishing unofficial Chinese media outlets, mostly owned by individuals and private companies, are comparable with traditional news media in the West in terms of function. The popular hosting platforms (e.g., WeChat, Sina Microblog, ZhiHu, and Bilibili) for the unofficial Chinese media outlets are also in and of itself a social media platform, making it very easy to share articles and comments to friends and families. Similar to how western traditional media also uses social media (e.g., Twitter and Facebook) to promote their news articles for views. Therefore, from this perspective, the comparison of how media affects individuals’ perception of events is valid in this context, though exceptions that disputable opinions or comments are restricted to access may still exist in these Chinese media outlets.

However, compared to North America and Western Europe, where more than half of the residents there have had actual experience with COVID-19 (35, 36), the low COVID-19 prevalence in China led to a greater likelihood that Chinese people obtain the information of COVID-19 through the media outlets (37, 38). Hence, Chinese people’s perception toward COVID-19 could be, to a much larger extent, dependent on unofficial reports of these media outlets, which have been found an effective medium to acquire relevant information for the public (37–39).

### The current study

Many Chinese owned media outlets frequently spread non-evidence-based information of COVID-19 or greatly exaggerated rare sequelae of Omicron lacking common consent of systematic study for the sake of attention, leading Chinese people to panic situation, in comparison with the large shift in attitudes toward Omicron in other countries around the world, especially in North America and Western Europe (40–42). Therefore, this article mainly intends to explore the association between Chinese people’s attitudes toward the potential sequelae of Omicron and their anxiety levels, as compared to these of people residing in North America or Western Europe. In addition, this study is also intended to present up-to-date information regarding risks of evidence-based sequelae that Omicron may cause to patients. Based on the backgrounds of COVID-19 and its information’s propagation *via* media outlets mentioned above in these countries, we propose Hypothesis 1 and Hypothesis 2.

1. Chinese people holding more negative attitudes toward the after-effects of Omicron will also have higher anxiety scores, compared to those residing in North America and Western Europe.
2. Residents in North America and Western Europe will have lower anxiety levels in terms of Generalized Anxiety Disorder 7-item (GAD-7) mean scores, compared to those residing in China.

## Materials and methods

### Overview

We conducted a questionnaire survey *via* the Credamo, a professional online survey platform similar to Qualtrics Online Sample (43), by randomly recruiting intending participants who were interested in our study, starting on June 1, 2022, and ending on June 8, 2022, and the use of human data from the surveys was carried out ethically in accordance with the principles of the Declaration of Helsinki (as revised in 2013). During this process, a Chinese version of questionnaire was used to collect the data from Chinese participants directly through a webpage-based answering platform on Credamo.^1^ An English version of the same questionnaire was separately submitted to the Credamo company to help collect the data from North America and Western Europe. To eliminate any potential misunderstanding of participants to questions due to different versions of questionnaires (i.e., Chinese vs. English), each question in the questionnaire was followed with a relevant example explaining the intention we were hoping to ask. On the first page of the questionnaire survey all participants received an adequate description of the purpose of the survey and were asked to tick a box to confirm an online informed consent prior to filling out survey. Furthermore, all data were collected anonymously through the Credamo using continuous identifier numbers to distinguish participants instead of recording their names or other sensitive information.

For survey answering quality, two attention check questions at different point in the survey were used. A one US dollar or RMB/GBP/Euro equivalent monetary incentive was offered for each participant who completed the survey. Meanwhile, we manually checked the time taken for completing each survey as well as the IP address of responders in case of the same responders joining the survey multiple times. Moreover, on the first page of the questionnaire survey, participants were informed about finishing the questionnaire truthfully under personal anxiety status developed explicitly during the pandemic era that Omicron dominated. Also, they were strictly informed that only those who did not experience any personal COVID-19 related situation that had caused their mental status deteriorated severely, were permitted to complete the questionnaire survey.

### Questionnaire contents

The questionnaire was mainly comprised of the following information collected:

1. Demographic information
2. How many shots of vaccine did you get?
3. Have you ever been infected with COVID-19?
4. Do you support coexistence or zero-tolerance approach of the Omicron-dominated pandemic in your country?
5. Do you have psychological fear toward your real-life friends who were infected with COVID-19 (i.e., do you want to be wary of them inwardly)?
6. Subjective attitudes toward Omicron about its after-effects (i.e., “to what extent do you think Omicron could cause sequelae?”)
7. The 7-item Generalized Anxiety Disorder-7 (GAD-7) scale.

A total of 1,500 people were initially recruited to complete the questionnaire survey. Invalid data were excluded, and new participants were recruited until 1,500 individual data fulfilled our standard inclusion criteria. Finally, a total of 1,623 people living in China, North America or Western Europe were independently recruited and surveyed through the Credamo platform. Of them, 78 were excluded for the failure in the attention check questions (e.g., responded wrongly to the instruction “please chose the answer Blue”), 33 were excluded for completing the survey in less than 100 s, 12 were excluded with additional analyses for other reasons such as answering the questionnaire questions inconsistently or contradictorily. Eventually, a valid sample of 1,500 participants was analyzed collectively (892 females and 192 males; mean age = 26.74 years, *SD* = 3.81 years; age range: 18–34 years). The effective response rate was 92.4%.

### Generalized anxiety disorder 7-item scale

The anxiety status of the participants was assessed using the 7-item version of the Generalized Anxiety Disorder scale or GAD-7. It consists of seven items based on seven core symptoms, asking respondents how often they experienced these symptoms in the past 2 weeks, and is preferably used to measure an individual’s proximate level of anxiety in a timely manner during the pandemic era (44–48). For each item, participants were asked to choose the degree to which they agreed or disagreed with the statement, on a scale of 0–3, with 0 denoting “not at all,” 1 denoting “several days,” 2 denoting “more than half the days” and 3 denoting “nearly every day.” In the GAD-7 scale, total score of participants for the seven items ranging from 0 to 21 was summed up. A total score of 0–4, 5–9, 10–14, and 15–21 were classified as minimal anxiety, mild anxiety, moderate anxiety, and severe anxiety, respectively. Hence, a higher total score indicated a higher level of anxiety status of participants. In this study, the Cronbach’s alpha coefficient for the total scale was 0.91, suggesting excellent overall internal consistency.

### Statistical analysis

All statistical analyses were performed using the software program SPSS (version 26.0) except for the data cleaning process which included detection and removal of invalid or missing data completed on the Credamo data platform. A reliability test was conducted for the GAD-7 scale, using Cronbach’s alpha coefficients as a measure of internal consistency (α > 0.70 regarded as acceptable). Mean differences were compared by using parametric tests. Finally, Kendall’s coefficient of rank correlation tau-sub-b was used to examine the association between the subjective attitudes of participants toward the after-effects of Omicron (ordinal variable) and the GAD-7 self-report scale scores (continuous variable), according to Khamis (49).

## Results

### Sample characteristics

There were 1,623 individuals from mainland China (Shanghai vs. non-Shanghai), North America and Western Europe, who enrolled in the survey, and 1,500 (92.4%) were included in the analysis participants after data cleaning. Relevant descriptive statistics were presented in Table 1.

**TABLE 1:** Sample description.

	*n* (%)		
*N* = 1,500	China (Shanghai) *n* = 500	China (non-Shanghai) *n* = 500	North America or Europe *n* = 500
**Variables**			
**Gender**			
Male	278 (55.6)	291 (58.2)	243 (48.6)
Female	222 (44.4)	209 (41.8)	257 (51.4)
**Vaccination status**			
1 dose	13 (2.6)	10 (2)	68 (13.6)
2 doses	28 (5.6)	110 (22)	105 (21.0)
3 doses	459 (91.8)	380 (76)	327 (65.4)
**Infection status**			
Yes	14 (2.8)	4 (0.8)	393 (78.6)
No	486 (97.2)	496 (99.2)	107 (21.4)
**View of coexistence with COVID-19**			
Support zero-tolerance approach (because of fearing sequelae of Omicron)	212 (42.4)	335 (67.0)	39 (7.8)
Support zero-tolerance approach (because of misgiving medical resource crowding)	93 (18.6)	79 (15.8)	20 (4)
Support co-existence with virus as much as possible	195 (39)	86 (17.2)	441 (88.2)
**Psychological fear toward friends infected with COVID-19**			
No	247 (49.4)	259 (51.8)	483 (96.6)
Yes	253 (50.6)	241 (48.2)	17 (3.4)
**Subjective attitude toward the sequelae of Omicron**			
No sequelae	5 (1.0)	11 (2.2)	283 (56.6)
Mild sequelae	54 (10.8)	229 (45.8)	106 (21.2)
Moderate sequelae	185 (37.0)	146 (29.2)	67 (13.4)
Severe sequelae	256 (51.2)	114 (22.8)	44 (8.8)

### Mean comparison of general anxiety disorder 7-item scores

**FIGURE 1:**
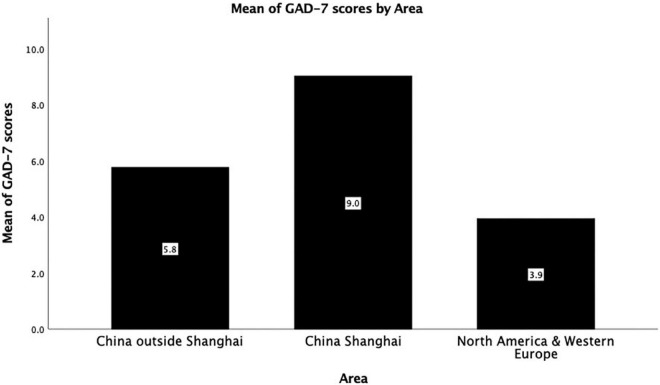
Mean of GAD-7 scores by area.

Regarding the mean differences of GAD-7 shown in Table 2, our results suggested that no any statistically significant difference was found in terms of gender, vaccination status, infection status of participants from outside Shanghai, view of coexistence with COVID-19, psychological fear toward friends infected with COVID-19 within groups (i.e., Non-Shanghai area (China), Shanghai (China), and North America and Western Europe; all *p* > 0.05), except for subjective attitudes of participants toward the sequelae of Omicron (all *p* < 0.05). However, there was strongly significant difference with reference to the mean of GAD-7 scores by area between groups as shown in Figure 1 (mean ± *SD* = 5.768 ± 3.59, 9.034 ± 3.93 and 3.94 ± 2.53, respectively; *F* = 287.485, *p* < 0.001).

**TABLE 2:** General anxiety disorder 7-item (GAD-7) scores of participants (*N* = 1,500).

Variables	GAD-7 scores								
	Means (*SD*)			[95%CI]			*p*		
	CN SH	CN Non-SH	NA and WE	CN SH	CN Non-SH	NA and WE	CN SH	CN Non-SH	NA and WE
Gender							0.578	0.731	0.664
Male	8.74 (3.97)	5.23 (2.34)	3.77 (1.80)	[8.27, 9.21]	[4.96, 5.50]	[3.54, 4.00]			
Female	9.11 (3.75)	6.03 (2.08)	4.11 (2.66)	[8.62, 9.60]	[5.75, 6.31]	[3.78, 4.44]			
Vaccination status							0.344	0.544	0.650
1 Dose	8.69 (3.97)	7.00 (3.33)	4.21 (2.59)	[6.53, 10.85]	[4.93, 9.07]	[3.59, 4.83]			
2 Doses	10.07 (4.81)	5.70 (3.71)	3.92 (2.57)	[8.29, 11.85]	[5.01, 6.39]	[3.43, 4.41]			
3 Doses	8.98 (3.87)	5.76 (3.56)	3.89 (2.52)	[8.63, 9.33]	[5.40, 6.12]	[3.62, 4.16]			
Infection status							0.000	0.837	0.067
Yes	4.06 (2.65)	10.50 (4.04)	3.89 (2.48)	[2.67, 5.45]	[6.54, 14.46]	[3.64, 4.14]			
No	7.07 (4.11)	5.73 (3.56)	4.15 (2.74)	[6.70, 7.44]	[5.42, 6.04]	[3.63, 4.67]			
VOC							0.936	0.092	0.099
SZAFS	9.17 (3.86)	5.63 (3.54)	4.73 (3.07)	[8.62, 9.72]	[5.25, 6.01]	[3.77, 5.69]			
SZAMM	8.93 (3.93)	7.16 (4.17)	4.64 (2.29)	[8.13, 9.73]	[6.24, 8.08]	[3.64, 5.64]			
SCV	9.35 (3.31)	6.05 (3.17)	4.50 (2.87)	[8.89, 9.81]	[5.38, 6.72]	[4.23, 4.77]			
PFTF							0.823	0.526	0.284
Yes	9.13 (3.86)	5.83 (3.54)	3.29 (2.14)	[8.65, 9.61]	[5.38, 6.28]	[2.27, 4.31]			
No	8.94 (4.00)	5.71 (3.64)	3.96 (2.55)	[8.44, 9.44]	[5.27, 6.15]	[3.73, 4.19]			
SATSO							0.000	0.000	0.060
No sequelae	6.80 (5.36)	3.64 (3.91)	3.95 (2.50)	[2.11, 11.49]	[1.33, 5.95]	[3.66, 4.24]			
Mild sequelae	7.04 (3.93)	4.64 (3.36)	3.45 (2.69)	[5.99, 8.09]	[4.20, 5.08]	[2.94, 3.96]			
Moderate sequelae	7.17 (2.73)	5.31 (2.34)	4.42 (2.23)	[6.78, 7.56]	[4.93, 5.69]	[3.89, 4.95]			
Severe sequelae	10.85 (3.79)	8.82 (3.61)	4.34 (2.68)	[10.39, 11.31]	[8.16, 9.48]	[3.55, 5.13]			

CN, China, SH, Shanghai, NA, North America; WE, Western Europe; VOC, View of coexistence with COVID-19; SZAFS, Support zero-tolerance approach (because of fearing sequelae of Omicron); SZAMM, Support zero-tolerance approach (because of misgiving medical resource crowding); SCV, Support co-existence with virus as much as possible; PFTF, Psychological fear toward friends infected with COVID-19; SATSO, Subjective attitude toward the sequelae of Omicron.

### Kendall’s tau-b analysis model

In Table 3, Kendall’s coefficient of rank correlation tau-sub-b as a type of inferential statistics was conducted to investigate the correlation between subjective attitudes of participants toward the sequelae of Omicron and their anxiety levels among different areas. Most importantly, it was found that subjective attitudes of Chinese participants toward the sequelae of Omicron were positively and significantly associated with their anxiety status (i.e., GAD-7 scores) in Shanghai (China) (*Tb* = 0.44, *p* < 0.01) and other parts of China outside Shanghai (*Tb* = 0.37, *p* < 0.01). However, no such significant correlation was found in North America & Western Europe (*Tb* = -0.01, *p* > 0.05). This meant that there was a tendency for Chinese participants to report higher levels of anxiety the more they feared the sequelae of Omicron.

**TABLE 3:** Correlation between SATSO and GAD-7 scores among different areas.

Kendall’s tau-b		SATSO in China (Shanghai) (*n* = 500)	SATSO in China outside Shanghai (*n* = 500)	SATSO in North America and Western Europe (*n* = 500)
GAD-7 scores	Correlation coefficient	0.44**	0.37**	−0.01
	Sig. (2-tailed)	0.00	0.00	0.70

N = 1,500. **Representing the correlation is significant at the 0.01 level (*p* < 0.01).

SATSO, Subjective attitude toward the sequelae of Omicron.

## Discussion

The given study mainly examined the association between participants’ attitudes toward the potential after-effects of Omicron and their anxiety status, and mean of GAD-7 scores of participants from different areas (Shanghai vs. Outside Shanghai within China vs. North America and Western Europe). The results of our study primarily showed that the more severe the sequelae of omicron the Chinese participants perceived, the higher their anxiety levels, but such relationship did not statistically and significantly exist in participants from North America and Western Europe. Also, regarding the mean of GAD-7 scores among different areas, participants from North America and Western Europe had relatively lowest anxiety levels (mean ± *SD* = 3.94 ± 2.53), followed by participants from China outside Shanghai (mean ± *SD* = 5.768 ± 3.59), and then participants from Shanghai (mean ± *SD* = 9.034 ± 3.93). These findings were consistent with our primary hypotheses in the introduction section. In addition, according to the results of one-way ANOVA as shown in Table 2, no statistical and significant difference of participants’ levels of anxiety was found in terms of gender, vaccination status, infection status of participants from outside Shanghai, subjective view of coexistence with COVID-19, personal psychological fear toward friends infected with COVID-19. However, there was a significant difference in terms of the infection status (Yes vs. No) of participants in Shanghai (China) and their corresponding GAD-7 mean scores. This might be understandable that the people in Shanghai were urgently required to respond a sudden pandemic situation, which led to a panic to the public with increased anxiety.

Admittedly, participants’ mindsets toward the pandemic might differ due to different cultures (50). But in the context of the COVID-19 pandemic in today’s advanced technological society, media outlets have been seen as useful means of spreading information about COVID-19 and measuring public attention toward COVID-19 in both China and the Western countries (37–39, 51, 52). Online COVID-19 infodemic (i.e., pandemic of misinformation), without prudent journalistic judgments of media content, could be easily and quickly disseminated and thus influence public opinions (39), therefore resulting in deadly consequences (51, 52). In addition, as we mentioned in the Introduction section, Chinese people’s perceptions toward COVID-19 could largely rely on the propagation of information of COVID-19 through media outlets, in comparison with the residents in North America and Western Europe, where a virus co-existence policy with relatively few restrictions to the public resulted in a great number of people being affected with COVID-19; but meanwhile, these people were thus allowed to have an actual experience of how long-term COVID-19 impacts their body, rather than only acquiring relevant information *via* media reports. Hence, the propagation of information about COVID-19 should be concerned, especially for the Chinese public.

Over the course of the COVID-19 pandemic, researchers are still struggling with the after-effects of coronavirus as it continued to evolve. Nowadays, as the COVID-19 variants prior to Omicron have nearly fade away, investigating and discussing the potential sequelae of Omicron that is the most prevalent variant in the current pandemic situation is necessary (53). Nevertheless, due to the significant lag in the publication of studies relevant to COVID-19 sequelae, the findings of recently published articles may not be applied to the latest Omicron situation. For example, the study suggesting that COVID-19 could lead to greater reduction in brain gray matter thickness was conducted in 2021 when the participants involved in this study were infected with the earliest variant of COVID-19 rather than Omicron (54); meanwhile, these participants were unvaccinated and generally older. Therefore, it is difficult to match these sequelae with the current less threatening Omicron. But a very recently published article suggested that the probability of Omicron causing long-term impacts to patients (4.5% of Omicron patients developed sequelae) was half that of Delta (10.8%) (55).

Some anecdotal findings reported by Chinese mass media indicated that most negative impacts of Omicron sequelae to human body were not reversible. However, that was not what previous studies actually found. For example, Zhao et al. suggested that mild Omicron sequelae such as slightly reduced attention and memory ability, which were even not perceived by participants themselves, were much improved over time (56). Similarly, another study followed the health status of patients with COVID-19 in Wuhan for 1 year after discharge from the hospital, and found that the after-effects of COVID-19 such as fatigue, sleeping disturbance and depression initially presented were improved greatly over time in these patients (57). Moreover, given that the study was conducted on the first batch of patients infected with COVID-19 in Wuhan, its findings were also not time-sensitive in the current context of Omicron.

Many anecdotal news online also stated that there was evidence that COVID-19 could induce male impotence. In fact, although a relevant study did suggest that COVID-19 may induce testicular damage, which could eventually result in decreased libido and fertility, the subjects involved in this study were animal rather than human patients; meanwhile, it was found that such negative impacts could be preventable by vaccination (58). However, when the findings of this study were reported by the mass media outlets, they overly exaggerated the impacts of COVID-19 by just saying “New study shows COVID-19 infection could cause testicular atrophy and reduced fertility in men.” The lack of evidence for statements such as the effects of COVID-19 on fertility and intelligence is not only unfair and discriminatory to those infected with COVID-19, but also affects the psychological state of those who have never suffered COVID-19 infection and increases their anxiety level. Therefore, the mass media reports were misleading to the public, which should have been avoided as much as possible. Regarding the effectiveness of vaccination against long COVID-19, two studies by Ayoubkhani et al. demonstrated that people who completed two doses of vaccine were less likely to develop long-term sequelae after being infected with COVID-19 (59, 60).

Furthermore, it is also important to note that the COVID-19 sequelae are not necessarily related to the COVID-19 itself. More specifically, any influenza or infectious disease may also induce similar negative impacts as COVID-19. For instance, a cross-sectional study with a large French cohort suggested that the so-called sequelae of COVID-19 perceived by patients themselves may be more psychological or actually caused by other diseases than the laboratory-confirmed result of COVID-19 infection (61).

Another issue to note is that our study found around half of the Chinese participants (Table 1) having psychological fear toward friends infected with COVID-19, though no significant difference between such mindsets and their anxiety levels was found in terms of GAD-7 mean scores. Therefore, we should advocate avoiding whether implicitly or explicitly discriminating people infected with COVID-19 who have the potential to suffer from various degrees of psychological disorders due to surrounding pressures such as social rejection.

Overall, with the widespread vaccination around the world, threats of the COVID-19 pandemic have been weakened. As can be seen from the outbreak in Shanghai recently, a large number of asymptomatic patients, even confirmed cases, were mainly mild symptom patients. Given that the global pandemic has become the norm, a total zero-out policy is not desirable. What we should do is to face the COVID-19 bravely with a more open and inclusive mind. In the current article, it seems that the fear of Omicron after-effects is more frightening than the COVID-19 itself in Chinese population. Thus, policy makers should enhance the public’s awareness of the latest change of pandemic situation, to eliminate unnecessary worries and reduce the psychological burden of Chinese people.

## Limitation

The current study has several limitations. First, this study was a cross-sectional study that might restrict causal inference. Second, the sample size was not large enough, thereby limiting the generalizability of this study. Third, this study was based on self-reported responses of participants. Although the data derived from an online professional data collection platform, more study with more professional research methods in similar topics is needed to carry out in the future, when conditions are permitted. Finally, another limitation in this study is the fact that participants’ media exposure was hard to track and measure, so a direct correlation between participants’ perceptions toward COVID-19 or Omicron specifically resulted from exposure to media outlets and their anxiety levels could be biased and still needs more study to further demonstrate.

## Conclusion

Currently, the global pandemic is subsiding as the novel coronavirus gradually evolves in a less harmful direction. However, due to the exaggeration about the long effects of Omicron by mass media outlets, which is currently the most prevalent variant of COVID-19, a variety of fears about Omicron long effects and a great deal of unpredictability about the future pandemic continue to plague people. In the current study we found that Chinese participants who were more worried about the after-effects of Omicron had higher levels of anxiety. Overall, although we still need to pay sufficient attention to COVID-19 and its long effects, we should take everything related to COVID-19 seriously based on the available scientific evidence, and not easily believe exaggerated or even false reports in the mass media. Also, to eliminate unnecessary worries and reduce the psychological burden of Chinese people, policy makers should put sufficient efforts to enhance the public’s awareness of the latest change of pandemic situation. In the future, more relevant studies are needed to reveal the long-term impacts of Omicron or subsequent variants of COVID-19.

## Data availability statement

The raw data supporting the conclusions of this article will be made available by the authors, without undue reservation.

## Ethics statement

Ethical review and approval were not required for this study on human participants in accordance with the local legislation and institutional requirements. The participants were notified to provide their written informed consent online before participation in this study.

## Author contributions

DS proposed the research idea, collected and analyzed the data from China, and wrote the initial manuscript. CL performed the literature search, provided the idea for the analysis, and collected the data from North America and Western Europe. SYL contributed to the substantial revisions of the manuscript in terms of reviewers’ comments. YDZ checked the accuracy of the data analysis and was responsible for final proofreading, contributing to minor revisions of the latest manuscript. All authors have read and approved the final version of the manuscript, and agree with the order of the presentation of the authors.

## Footnotes

1
www.credamo.com
