# Corrigendum: Increased anxiety from fear of Omicron in China as compared to North America and Western Europe: A cross-sectional Kendall's tau-b analysis using the generalized anxiety disorder 7-item questionnaire

**DOI:** 10.3389/fpsyt.2022.1038966

**Published:** 2022-10-06

**Authors:** Dan Shan, Chang Liu, Shaoyang Li, Yuandian Zheng

**Affiliations:** ^1^School of Medicine, National University of Ireland, Galway, Ireland; ^2^School of Clinical Medicine, University of Cambridge, Cambridge, United Kingdom; ^3^Faculty of Science, The Hong Kong Polytechnic University, Hong Kong, Hong Kong SAR, China; ^4^College of Osteopathic Medicine, Kansas City University, Kansas City, MO, United States

**Keywords:** anxiety, COVID-19, Omicron, pandemic, sequelae

In the original article, there was a statistical error in the **Materials and Methods** section, page 4. The text read as “Eventually, a valid sample of 1,500 participants was analyzed collectively (892 females and 192 males; mean age = 26.74 years, SD = 3.81 years; age range: 18–34 years).” The statement has been corrected as follows:

“Eventually, a valid sample of 1,500 participants was analyzed collectively (688 females and 812 males; mean age = 26.74 years, SD = 3.81 years; age range: 18–34 years).”

The authors apologize for this error and state that this does not change the scientific conclusions of the article in any way. The original article has been updated.

## Publisher's note

All claims expressed in this article are solely those of the authors and do not necessarily represent those of their affiliated organizations, or those of the publisher, the editors and the reviewers. Any product that may be evaluated in this article, or claim that may be made by its manufacturer, is not guaranteed or endorsed by the publisher.

