# Correction: Increased anxiety from fear of Omicron in China as compared to North America and Western Europe: a cross-sectional Kendall’s tau-b analysis using the generalized anxiety disorder 7-item questionnaire

**DOI:** 10.3389/fpsyt.2026.1893139

**Published:** 2026-06-04

**Authors:** Dan Shan, Chang Liu, Shaoyang Li, Yuandian Zheng

**Affiliations:** 1 COVID-19 Response Social Organization Collaboration Network, Qujing, China; 2 School of Clinical Medicine, University of Cambridge, Cambridge, United Kingdom; 3 Faculty of Science, The Hong Kong Polytechnic University, Hong Kong, Hong Kong SAR, China; 4 College of Osteopathic Medicine, Kansas City University, Kansas City, MO, United States

**Keywords:** anxiety, COVID-19, Omicron, pandemic, sequelae

Affiliation “COVID-19 Response Social Organization Collaboration Network, Qujing, China” was erroneously given as “School of Medicine, National University of Ireland, Galway, Ireland” for author Dan Shan.

The original version of this article has been updated.

